# Zone V Extensor Tendon Repair with a Palmaris Longus Tendon Autograft and Human Umbilical Membrane

**DOI:** 10.1155/2020/2759281

**Published:** 2020-01-03

**Authors:** Esteban Esquivel, Cameron Cox, Amanda Purcell, Brendan MacKay

**Affiliations:** Department of Orthopaedic Surgery and Rehabilitation, Texas Tech University Health Sciences Center, Lubbock, TX, USA

## Abstract

Extensor tendon repairs, although common, can be difficult injuries to treat. Their treatment is tailored to the zone of the hand that is affected since varying biomechanical forces are applied to the tendon at each zone. Prompt treatment is necessary to prevent potential complications associated with these injuries. This is particularly true of Zone V extensor tendon injuries, as their mechanism is commonly a highly infectious human bite. We present the case of a human fight bite resulting in a Zone V extensor tendon injury. The delayed presentation of this case resulted in an untreated infection that caused an abscess with associated extensor tendon necrosis and rupture. Given the large gap length between the ends of the tendons, tendon repair was performed using a palmaris longus autograft. Even when these are done in a controlled setting, adhesions are common. The compromised wound bed caused irritation, erosion, and subsequent rupture of the extensor tendon of the hand. In an effort to avoid common complications such as adhesion, the repair was then wrapped with human umbilical membrane (AVIVE® Soft Tissue Membrane, AxoGen Inc., Alachua, FL) to separate adjacent tissue and reduce inflammation. Even without access to formal physical therapy, our patient had excellent functional outcomes at his final follow-up visit. The patient was able to make a loose composite fist, had no extensor lag at the MCP joints, and had extensor lag of 15 degrees at the PIP joints of digits 4-5.

## 1. Introduction

Extensor tendon injuries are more common than flexor tendon injuries and account for more than half of all acute tendon traumas, with an incidence rate of 14 cases per 100,000 person-years [1]. This may be in part due to the anatomy of the extensor tendons and surrounding tissue including their superficial location, flatter profile, and closer proximity to bony structures. Due to their reduced size and absence of collagen-bundle linkage, extensor tendons also provide less grip strength for suture placement [2].

Generally, the three management options available for extensor tendon injuries involve either nonoperative management, primary repair, or tendon grafting. Treatment of these injuries depends primarily on the zone of the hand involved and should be done within a reasonable time frame as deformities, restricted range of motion, and disseminated infection can occur if left untreated. For example, a closed Zone I injury can be managed with splinting alone whereas a Zone II injury with a tendon laceration greater than 50% will require primary repair followed by splinting [1].

When possible, Zone V extensor tendon injuries should be treated with primary tendon and sagittal band repair as needed [3]. Cases in which the extensor tendon is not amenable to primary repair may require a tendon graft for reconstruction. The palmaris longus tendon is often used as a tendon graft for reconstruction of tendons and ligaments in the body because of its length and diameter [4, 5]. The palmaris longus is considered an accessory muscle as it is missing in approximately 13% of people and does not cause reduced function if absent [4, 5]. While its use has been reported in Zone 4 repairs, we were unable to find a case of Zone 5 repair using palmaris longus in the literature [6].

A compromised wound bed is a poor prognostic factor for tendon repair in the hand. While the literature is lacking on extensor tendon repairs with concomitant infection, it has been reported in flexor tendon injuries. One case series found that an infected wound bed was significantly associated with poor results, with 0% of infected repairs resulting in good or fair functional outcomes as measured by the Strickland classification [7].

We present the case of a Zone V injury to the extensor tendon of the hand resulting from what is often referred to as a “fight bite.” Subsequent infection required irrigation, debridement, and ultimately reconstruction of the tendon. The extensor tendon gap was bridged using an autologous palmaris longus graft, and the repair was wrapped with AVIVE® Soft Tissue (AxoGen Inc., Alachua, FL) Membrane to separate the repair from adjacent tissue and reduce inflammation.

## 2. Case Presentation

A 37-year-old male presented with a laceration to the dorsal aspect of his right hand and 4^th^ digit. Six days prior to admission, the patient was involved in an altercation where he struck an individual in the mouth. Over several days, the patient developed severe, constant pain, limited mobility of the 4^th^ digit, swelling, erythema, and purulent drainage.

Six days after the injury, the patient presented to the emergency room and had a 2 cm laceration to the dorsum of the right hand at the level of the 4^th^ MCP joint with gross purulence and a foul odor. The patient was unable to extend the 4^th^ and 5^th^ digits from the flexed position. Extensors and flexors of the thumb appeared intact along with the intrinsic muscles of the hand. Sensation to light touch was intact in the distributions of the axillary, radial, ulnar, and median nerves, and all digits had brisk capillary refill.

**Figure 1:**
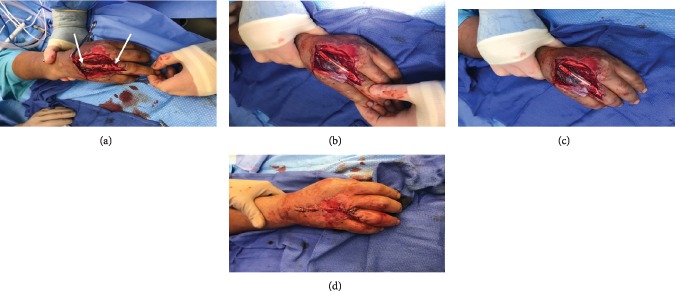
Intraoperative images of Zone V extensor tendon repair of the right hand. (a) Rupture of extensor tendon at Zone V. (b, c) Avive® Soft Tissue Membrane wrapped around palmaris longus tendon autograft. (d) Closure of incision with staples.

The patient underwent surgery for irrigation and debridement, as well as exploration and potential repair of damaged structures. The traumatic laceration was extended proximally and distally to approximately 7 cm. Once in the 4^th^ MCP joint, purulent fluid was noted and cultures were taken. There was necrotic tissue, and the infection had eroded through the extensor tendon causing rupture in Zone V (Figure 1(a)). The patient was started on broad spectrum IV antibiotics, and a second surgery was planned in 48 hours.

After further irrigation and debridement, the compromised tissue was resected back to reveal a 10 cm gap in the extensor tendon, which would necessitate reconstruction. Intraoperative cultures grew *Streptococcus anginosus* and *Eikenella corrodens*.

Six days after initial presentation, the patient was taken to the operating room for a third time. At this time, there was no progression of the infection, and the wound bed appeared to be stable with minimal need for debridement. Given the 10 cm gap, the decision was made to use palmaris longus autograft. This was harvested using standard technique with two transverse incisions at the proximal and distal ends of the forearm. The graft was connected to each end of the ruptured extensor tendon using a Pulvertaft weave.

A 3 × 6 cm piece of AVIVE® Soft Tissue Membrane was wrapped around the tendon reconstruction and secured with a chromic suture (Figures 1(b) and 1(c)). The wound was closed with chromic sutures and staples (Figure 1(d)), dressed, and the patient was placed in a volar resting splint for the entire hand to the PIP joints. This provided support and prevented flexion and/or extension beyond what the repair could sustain within the first week.

## 3. Outcome

The patient was seen in the clinic 1 week postoperatively. At this time, there was found to be no recurrence of infection, and he only had moderate edema of the dorsal hand and digits. Due to the patient's lack of access to physical therapy, he had to be taught ROM exercises including active metacarpophalangeal (MP) flexion and gentle passive extension for 10 repetitions every hour. He was given a splint that was fabricated in the office rather than the standard thermoplastic splint typically given at therapy to block the MCPs of digits 3-5.

Due to his limited access to transportation, the patient failed to appear at his 2- and 4-week appointments.

Normally, patients with this injury pattern begin active metacarpophalangeal (MP) flexion exercises up to 30-40° using a flexion block on a dynamic splint 1-3 days postop. At this time, passive extension is also initiated by the elastic force of the dynamic splint, and 10 repetitions are completed per hour. Both exercises are continued for 3 weeks with progressing ROM as tolerated [8].

MP flexion is typically increased to 40-60 degrees by the end of week 4 and 70-80 degrees by week 5. During the 4-6-week period, full fisting is initiated as well as composite wrist and active digital extension without the splint. At 6 weeks, active assisted range of motion (AAROM), stretching, progressive resistance exercises, and reverse putty scraping are initiated. At this time, patients are typically fitted with a dynamic flexion splint [8].

While he remained unable to attend therapy, the patient did make his 6-week follow-up appointment. At this time, he had no extensor lag of the MCP joints, had extensor lag of 30 degrees at the PIP joints of digits 4-5, and was unable to make a composite fist due to stiffness as he had not been able to attend therapy sessions.

The patient was given PO Methylprednisolone to alleviate edema and reeducated in the rehab protocol for extensor tendons. The patient stated that he could not afford therapy or further splinting and was shown how to perform AAROM, stretching, progressive resistance, and putty scraping exercises at home.

**Figure 2:**
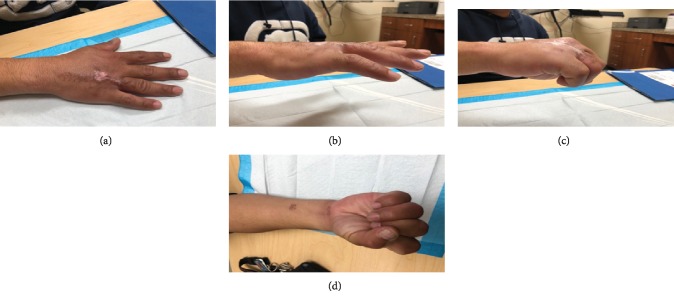
Postoperative images taken at 3 months postop following Zone V extensor tendon repair of the right hand. (a) Extension of digits on flat surface. (b) No extensor lag at MCP joints and extensor lag of 15 degrees in digits 4-5 at PIP joints. (c, d) Flexion of digits intact, limited due to stiffness.

At 3 months postop, no extensor lag was noted at the MCP joints and extensor lag of 15 degrees was present at the PIP joints of digits 4-5 (Figures 2(a) and 2(b)). Furthermore, the patient was able to make a loose composite fist (Figures 2(c) and 2(d)).

## 4. Discussion

In the United States, an estimated 4.5 million dog bites, 400,000 cat bites, and 250,000 human bites occur each year, with the hand being the most common site for bite injuries [9]. Even though they are less common, human bites are associated with infection rates of 20-25% [10]. Without treatment, pathogens from the human oral flora may proliferate in the open wound and cause infection [10].

Human bites to the hand are often the result of a fist contacting a tooth in an altercation—a fight bite. Fight bites are the most common mechanism of Zone V extensor tendon injuries. Due to the increased risk of infection presented by this injury pattern, it is crucial to start irrigation, debridement, and antibiotic therapy as quickly as possible [3].

One complicating factor in the treatment of fight bites is their often delayed presentation. Delayed treatment of these injuries has been associated with complications including septic arthritis, osteomyelitis, and amputation in some cases [11]. In a review of 124 patients, with human bite injuries to the hand, only 38% presented within 24 hours of the injury, all of which had excellent outcomes. In patients who presented 1-7 days after the injury, outcomes were largely dependent on the presence of infection. Those without infection had good outcomes, but those with infection had decreased strength and range of motion [12]. A separate case series found that patients presenting within 48 hours of the initial injury had superior outcomes compared to those of late presentation [13].

While the literature on complication rates in extensor tendon injuries is limited, there have been many reports on flexor tendon repairs. One meta-analysis evaluated 20 major reports over the course of 15 years to track outcomes of flexor tendon repairs in the hand. They found that 4-10% of repairs ruptured, and 10% developed restrictive adhesion leading to tenolysis or secondary grafting [14]. Once tendon adhesion has developed, it is unlikely that the patient will achieve excellent range of motion. One study reported only 31% improvement in total active motion from tenolysis performed after extensor tendon repair [15].

Given the common occurrence of adhesion in extensor tendon repairs, multiple materials have been used to limit the formation of adhesions postrepair. Hyaluronic acid (HA) has been used in various tendon repairs to reduce adhesions and inflammation for improved tendon gliding. Current studies on HA have utilized animal models, and short-term clinical results have been promising [16]. Interferon-alpha, polymers, and collagen-elastin matrices have also been used to prevent adhesions, but none have had reproducible success. Recently, amniotic membranes have shown clinical utility in preventing frequent complications of tendon repair [17].

The amniotic membrane was first reported for its use as a biological scaffold in the early 1900s and has since gained popularity as a dressing and as a regenerative template. Amniotic membrane products are known to reduce scar formation by utilizing hyaluronic acid present in the mesenchymal layer to inhibit fibroblast activation [18, 19]. Interleukin-10 in the epithelial cells of amniotic tissue suppresses the expression of proinflammatory cytokines and antigens to reduce inflammation [20]. In addition to their antiscarring and anti-inflammatory properties, growth factors within amniotic mesenchymal and epithelial cells promote the proliferation and differentiation of epithelial cells [18, 20].

There are many amniotic membrane products currently used in orthopedics. These are available in particulate form as well as in sheets, wraps, and liquids. Their uses include wound coverage, soft tissue regeneration, and acting as a barrier between tissues in nerve and tendon repairs [19].

Research on the use of the amniotic membrane in human tendon injuries has been limited to injectable forms. One study reviewed outcomes for 10 patients injected with the fluid placental tissue matrix for lower-extremity tendon injuries. 100% of these patients reported no pain at the 5-week follow-up [21]. A separate case series evaluated 44 patients receiving amniotic tissue fluid injection after failing standard treatment for 6 months. At the 12-week follow-up, all patients experienced temporal reduction in the pain score, and all reported VAS pain scores of <4 scores [21].

Another study followed 40 patients (20 with tendinosis and 20 with arthropathic conditions) who had received amniotic tissue injections to assess changes in the pain level, daily activities, and sports activities up to 3 months after procedures. At 3 months, the average pain score had decreased to 1.4, and 91% of patients achieved more than 30% improvement in function [21].

The literature addressing amniotic membrane sheet use in tendon repair is limited to animal models. In a recent 2019 article, type II diabetic model rates incurred a full-thickness tendon laceration which was then directly repaired and wrapped with human dehydrated amnion/chorion membrane (dACM). The use of dACM resulted in improved biomechanical properties, increased cell migration, and upregulation of important growth factors [22].

AVIVE® Soft Tissue Membrane is a processed human umbilical cord amniotic membrane that is commercially available in sheet form. Because it is harvested from the umbilical cord rather than the amniotic sac, AVIVE® is 8 times thicker than other available amniotic membranes. This improves durability, with suture pullout strength of 1.47 N in the umbilical membrane compared to 0.45 N in the amnion-chorion membrane. The thicker umbilical membrane persists up to 16 weeks and is easy to handle intraoperatively [20].

AVIVE® is most commonly used in nerve repairs. Two ongoing studies have reported promising preliminary results in wrapping nerve repairs in humans [23, 24]. One of these reported postoperative reduction in pain scores from 4.3 to 2.4 on average [24]. While it is commonly used to wrap nerves, AVIVE® is indicated for use broadly as a soft tissue covering [20, 25].

Considering the delayed presentation of an open wound with an active infection, one would have expected a poor outcome with extensive tendon adhesion development following extensor tendon repair. Even in uncomplicated repairs, it has been reported that as many as 10% of patients fail to reach good or excellent results in repairs of Zones 5-8 [26]. The literature suggests that dynamic splinting and early postoperative range of motion therapy play a large role in reducing complications. Recently, early mobilization protocols for extensor tendon repairs have been developed in efforts to improve outcomes. The use of early mobilization and dynamic splints has yielded a higher percentage of good and excellent outcomes and increased active range of motion [27, 28]. Yet, in cases where only static splinting is used, 17-30% of patients still required secondary tenolysis [29].

Even without access to physical therapy, and being unable to attend regular follow-up visits, our patient did not experience any of the aforementioned complications. AVIVE® Soft Tissue Membrane was used successfully in this extensor tendon repair to limit inflammation, prevent adhesion, and stimulate soft tissue regeneration. Our patient had excellent function at the MCP joint for flexion and extension following repair. He also had excellent flexion and a 15-degree extensor lag at the PIP joint [30]. This outcome was satisfactory to the patient and is favorably aligned with expectations outlined by Newport et al. and, more recently, Mehdinasab et al. who evaluated the outcomes of extensor tendon injuries at various zones of the hand [31, 32]. Newport et al. found excellent results in 83.3% of Zone V injuries following primary repair while Mehdinasab et al. found excellent results in 53.8% of Zone V injuries [31, 32].

## 5. Conclusion

The delayed presentation of this case resulted in an untreated infection that caused irritation, erosion, and subsequent rupture of the extensor tendon of the hand. This case report suggests that repair with a palmaris longus tendon autograft is a viable option for treatment of Zone V extensor tendon rupture and that AVIVE® Soft Tissue Membrane may be used as an adjunct in treating tendon injuries of this type. Further studies with a larger cohort should be performed to assess the outcomes for Zone V extensor tendon repairs using this approach.
